# Effect of Endothelial Culture Medium Composition on Platelet Responses to Polymeric Biomaterials

**DOI:** 10.3390/ijms22137006

**Published:** 2021-06-29

**Authors:** Skadi Lau, Anna Maier, Steffen Braune, Manfred Gossen, Andreas Lendlein

**Affiliations:** 1 Institute of Active Polymers and Berlin-Brandenburg Center for Regenerative Therapies, Helmholtz-Zentrum Hereon, 14513 Teltow, Germany; skadi.lau@hereon.de (S.L.); anna.maier.berlin@gmail.com (A.M.); steffen.braune@b-tu.de (S.B.); manfred.gossen@hereon.de (M.G.); 2 Institute of Chemistry, University of Potsdam, 14476 Potsdam, Germany

**Keywords:** cyclic olefin copolymer, poly(tetrafluoroethylene), endothelial cells, platelets, in vitro thrombogenicity testing

## Abstract

Near-physiological in vitro thrombogenicity test systems for the evaluation of blood-contacting endothelialized biomaterials requires co-cultivation with platelets (PLT). However, the addition of PLT has led to unphysiological endothelial cell (EC) detachment in such in vitro systems. A possible cause for this phenomenon may be PLT activation triggered by the applied endothelial cell medium, which typically consists of basal medium (BM) and nine different supplements. To verify this hypothesis, the influence of BM and its supplements was systematically analyzed regarding PLT responses. For this, human platelet rich plasma (PRP) was mixed with BM, BM containing one of nine supplements, or with BM containing all supplements together. PLT adherence analysis was carried out in six-channel slides with plasma-treated cyclic olefin copolymer (COC) and poly(tetrafluoro ethylene) (PTFE, as a positive control) substrates as part of the six-channel slides in the absence of EC and under static conditions. PLT activation and aggregation were analyzed using light transmission aggregometry and flow cytometry (CD62P). Medium supplements had no effect on PLT activation and aggregation. In contrast, supplements differentially affected PLT adherence, however, in a polymer- and donor-dependent manner. Thus, the use of standard endothelial growth medium (BM + all supplements) maintains functionality of PLT under EC compatible conditions without masking the differences of PLT adherence on different polymeric substrates. These findings are important prerequisites for the establishment of a near-physiological in vitro thrombogenicity test system assessing polymer-based cardiovascular implant materials in contact with EC and PLT.

## 1. Introduction

Polymer-based cardiovascular implant materials such as expanded poly(tetrafluoro ethylene) (ePTFE) are prone to platelet-mediated thrombosis formation followed by graft stenosis or total occlusion (reviewed in [1]). In native vessels, endothelial cells (EC) are primarily responsible for the maintenance of an undisturbed blood flow by preventing platelet (PLT) adherence. Thus, endothelialization of blood-contacting implant materials is considered a promising strategy to reduce the risk of thrombosis. However, a phenotypically dense endothelial monolayer per se is not equivalent to anti-thrombogenic functions of EC. For the latter, EC have to be in a functional state that is sometimes referred to as functionally confluent [2,3]. To check this, EC and PLT need to be co-cultivated. To date, only few studies considered such co-cultures in hemocompatibility assays, primarily because suitable in vitro test systems for the co-cultivation of EC and PLT are missing [4]. One of the challenges reported is the influence of PLT on adherent EC, which leads to the detachment of EC [5]. The underlying reasons for this phenomenon are unknown. This explains why those studies, which included co-culture experiments, often used additives such as prostacyclin or acetylsalicylic acid to prevent PLT adherence to EC [6,7]. Alternatively, PLT were washed prior to co-cultivation to remove membrane proteins responsible for PLT adherence [8]. However, this does not reflect the physiological situation. Multiple factors might be responsible for the detachment of EC such as the endothelial cell medium, which is specifically made for EC rather than PLT. PLT are highly sensitive and react to numerous mechanical or biochemical stimuli with adherence, activation, and aggregation. Thus, to investigate the hemocompatibility of endothelialized biomaterials in vitro, it is necessary to identify culture conditions that do not activate PLT per se. One of the most important parameters potentially confounding a meaningful co-cultivation experimental set-up is the use of endothelial culture medium, which is much more complex in its composition compared to media for other cell types [9]. As some of these ingredients such as insulin-like growth factor 1 and heparin are known to activate PLT, it can be speculated that other factors might do so as well [10,11]. Aiming at the establishment of a near-physiological in vitro test system suitable to investigate the thrombogenicity of new polymer-based implant materials, it was hypothesized that individual endothelial cell medium components induce PLT responses. To verify this hypothesis, a commonly used endothelial cell culture medium was systematically analyzed to elucidate the possible effect of individual media components on PLT adherence, activation, and aggregation. Assessment of PLT adherence was performed under static conditions using channel slides with cyclic olefin copolymer- or poly (tetrafluoro ethylene)-based substrates in the absence of EC. It is noteworthy that the channel walls and ceiling are composed of a second type of polymer (cyclic olefin polymer), which might also influence PLT responses. PLT activation and aggregation was analyzed in the absence of these two biomaterials.

## 2. Results

### 2.1. Platelet Adherence

**Figure 1:**
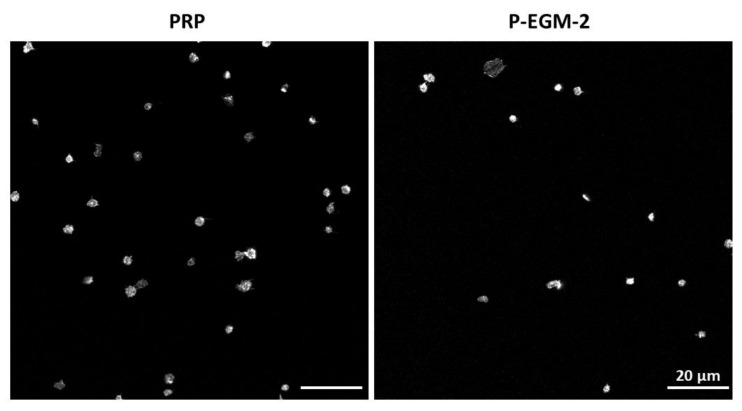
Adherence of platelets (PLT) on plasma-treated cyclic olefin copolymer (pCOC). Platelet-rich plasma (PRP) was incubated under static conditions for 1 h at 37 °C with basal medium containing nine supplements (P-EGM-2) in a ratio of 1:1 resulting in a concentration of 50,000 PLT µL^−1^. PRP served as control. PLT of six different donors were stained with phalloidin conjugated with Alexa Fluor 555 and images were taken by confocal laser scan microscopy at 100-fold primary magnification. As platelet adherence was similar for all six donors and for all supplements tested, representative images of PLT of one donor are shown for the complete medium (P-EGM-2) and for the control PRP. Scale bar represents 20 µm.

**Figure 2:**
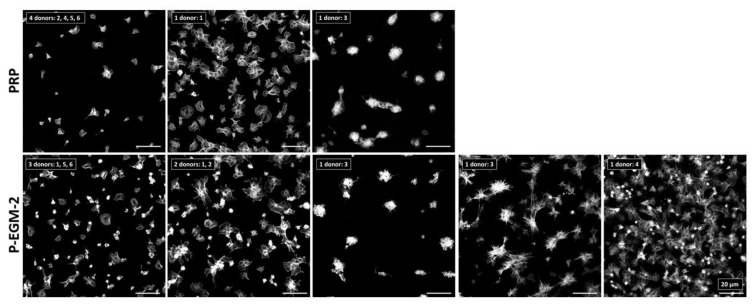
Adherence of platelets (PLT) on poly(tetrafluoro ethylene). Platelet-rich plasma (PRP) was incubated under static conditions for 1 h at 37 °C with basal medium containing nine supplements (P-EGM-2) in a ratio of 1:1 resulting in a concentration of 50,000 PLT µL^−1^. PRP served as control. PLT of six different donors (numbered from 1–6) were stained with phalloidin conjugated with Alexa Fluor 555 and images were taken by confocal laser scan microscopy at 100-fold primary magnification. Shown are different classes of platelet morphologies (with the individual donors representing them as indicated) after incubation with the complete medium (P-EGM-2; 5 different classes) or without any medium components (PRP; 3 different classes). For example, PLT of four donors (No. 2, 4, 5 and 6) incubated with PRP adhered in low numbers and show a spread morphology. Scale bar represents 20 µm.

To study the effect of different compositions of endothelial cell culture media on platelet adherence, PLT were incubated with each of the 11 compositions listed in Table 1 for 1 h under static conditions using six-channel slides with either pCOC or PTFE as a substrate. On pCOC, PLT from nearly all investigated donors (*n* = 6) showed a similar morphology independent of the supplement used (Figure 1; PLT morphologies in response to the remaining supplements can be seen in Supplementary Figure S1). Most adherent PLT exhibited a compact and discoid morphology while few PLT incubated with the complete endothelial cell medium (P-EGM-2) were spread-discoid. All PLT were clearly separated from each other. Representative images are shown for PRP, which served as control, and the complete endothelial cell medium, which displays all morphologies that were observed for PLT in contact with individual supplements. In contrast to pCOC, more PLT adhered to PTFE, which is a highly thrombogenic material. Moreover, they displayed different morphologies that can be attributed to different activation states of PLT. Generally, the morphology of PLT after incubation with different supplements was diverse and clearly donor-dependent. Therefore, two or more images are shown for PRP and for PLT incubated with P-EGM-2, which represent all morphologies that were observed after incubation of PLT with individual supplements (Figure 2). In case of PRP, three different morphologies of PLT were detected. PLT of most donors adhered in low numbers and showed a spread morphology (donors: 2, 4, 5, 6). In contrast, PLT of donor 1 adhered in high numbers and were spread-discoid in shape. PLT of donor 3 formed many cohesive aggregates on PTFE (Figure 2, upper row). The incubation of PRP with the complete medium (P-EGM-2) led to five different classes of morphology. While PLT of half of the tested donors displayed spread-discoid PLT that were distantly located from each other, PLT from two donors were slightly branched and showed pseudopodia formation. PLT of donor 3 showed two different morphologies such as compact and cohesive PLT aggregates, as well as a star-like morphology. Finally, PLT of donor 4 showed an advanced activation state with highly branched PLT lying on top of each other and pseudopodia formation (Figure 2, lower row; PLT morphologies in response to the remaining supplements can be seen in Supplementary Figure S2).

To quantitatively assess PLT adherence in dependence of EC medium compositions on pCOC and PTFE, the mean area per platelet, the platelet covered area and the number of adherent platelets were determined.

**Figure 3:**
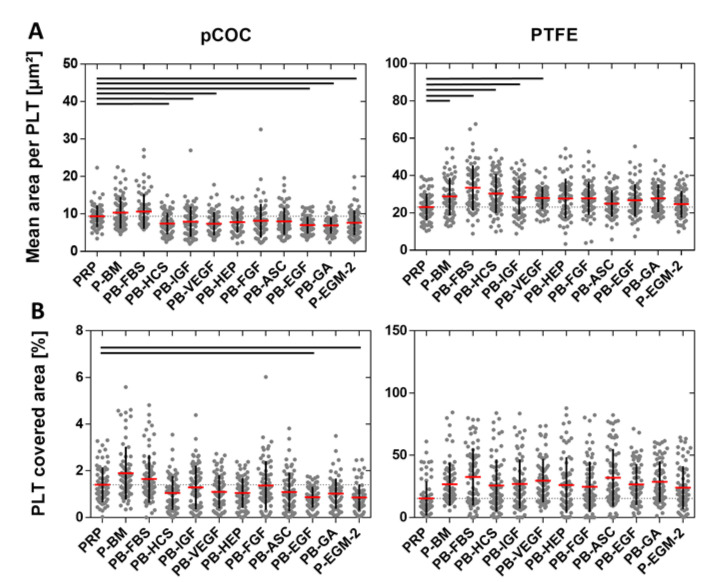
Quantification of platelet adherence to plasma-treated cyclic olefin copolymer (pCOC) and poly(tetrafluoro ethylene) (PTFE) assessed as mean area per platelet (PLT) and PLT covered area. Platelet-rich plasma (PRP) was incubated under static conditions for 1 h at 37 °C with basal medium (BM) or with BM containing one of nine different supplements or with BM containing all nine supplements (see Table 1 for nomenclature) in a ratio of 1:1 resulting in a concentration of 50,000 PLT µL^−1^. PRP served as control. Images of phalloidin-stained PLT were quantitatively analyzed by ImageJ software to obtain the mean area per PLT (**A**) and the PLT covered area (**B**). Thin dashed line shows the value for PRP (control). Data represent the arithmetic mean (red) ± standard deviation (black), *n* = 72 per supplement. Black lines indicate significant differences with *p* ≤ 0.0493.

The mean area per platelet was significantly larger on PTFE (28 ± 3 µm^2^) than on pCOC (8 ± 1 µm^2^; *p* ≤ 0.0001). On pCOC, a significantly smaller mean area per PLT was detected for PB-HCS, PB-IGF, PB-VEGF, PB-EGF, PB-GA, and P-EGM-2 (ranging from 7 ± 2 µm^2^ to 8 ± 4 µm^2^; *p* ≤ 0.0493) compared to PRP (9 ± 3 µm^2^). In contrast, incubation with PB-BM and PB-FBS led to a larger mean area per PLT (PB-BM: 10 ± 4 µm^2^ and PB-FBS: 11 ± 5 µm^2^), but this tendency was not significant. On PTFE, the addition of all supplements to PLT resulted in an increased mean area per PLT in comparison to PRP (23 ± 7 µm^2^); however, significant differences were detected only for PB-BM, PB-FBS, PB-HCS, PB-IGF, and PB-VEGF (ranging from 28 ± 6 µm^2^ to 33 ± 12 µm^2^; *p* ≤ 0.0307) (Figure 3A).

The PLT covered area is another parameter for the assessment of thrombogenicity. While on pCOC, only 1.2 ± 0.3% of the area was covered by PLT, a considerably larger area of 26.5 ± 4.2% was covered by PLT on PTFE (*p* ≤ 0.0001). In particular, on pCOC, the PLT covered area after incubation with PRP (1.4 ± 0.7%) was larger than with all supplements apart from PB-BM and PB-FBS. However, these differences were only significant in case of PB-EGF (0.9 ± 0.5%; *p* = 0.0028) and P-EGM-2 (0.9 ± 0.6%; *p* = 0.0005). In contrast, on PTFE, the PLT covered area was significantly increased after contact of PLT with all supplements (ranging from 24.0 ± 17.2% to 32.5 ± 22.8%; *p* ≤ 0.0475) compared to PRP (15.3 ± 13.7%) (Figure 3B). 

**Figure 4:**
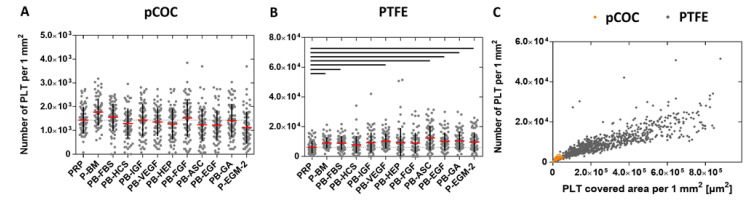
Polymer-dependent promotion of platelet (PLT) adherence. Platelet-rich plasma (PRP) was incubated under static conditions for 1 h at 37 °C with basal medium (BM) or with BM containing one of nine different supplements or with BM containing all nine supplements (see Table 1 for nomenclature) in a ratio of 1:1 resulting in a concentration of 50,000 PLT µL^−1^. PRP served as control. Images of phalloidin-stained PLT were quantitatively analyzed by ImageJ software. Shown are the number of adherent PLT on COC (**A**) and PTFE (**B**) as well as the relationship between PLT number and PLT covered area (**C**). Thin dashed line shows the value for PRP (control). Data represent the arithmetic mean (red) ± standard deviation (black), *n* = 72 per supplement. Black lines indicate significant differences with *p* ≤ 0.0475. A comparison of the two substrates regarding thrombogenicity shows that PTFE (grey rhombuses) is more thrombogenic than COC (orange circles; C). *n* = 864 for each polymer.

The number of adherent PLT, which increases in response to pro-thrombogenic conditions, is also an important parameter to assess the thrombogenicity of biomaterials. Here, considerably more PLT adhered to PTFE (9300 ± 1500 PLT mm^−2^) than to pCOC (1400 ± 200 PLT mm^−2^, *p* ≤ 0.0001). On pCOC, none of the tested supplements had an effect on the number of adherent platelets in comparison to PRP (Figure 4A). In contrast, on PTFE, significantly more PLT adhered after contact with PB-BM, PB-FBS, PB-VEGF, PB-ASC, PB-EGF, PB-GA, and P-EGM-2 (ranging from 8900 ± 4600 PLT mm^−2^ to 12,300 ± 8100 PLT mm^−2^, *p* ≤ 0.0368) compared to PRP (6100 ± 4200 PLT mm^−2^) (Figure 4B). Figure 4C illustrates the relationship between the PLT number and PLT covered area, which are both reduced on pCOC compared to PTFE. 

In summary, these data show that PLT adherence in the presence of different supplements is polymer- and donor-dependent. Moreover, PTFE is much more thrombogenic than pCOC and PLT adherence on PTFE is much more variable than on pCOC.

### 2.2. Platelet Activation

**Figure 5:**
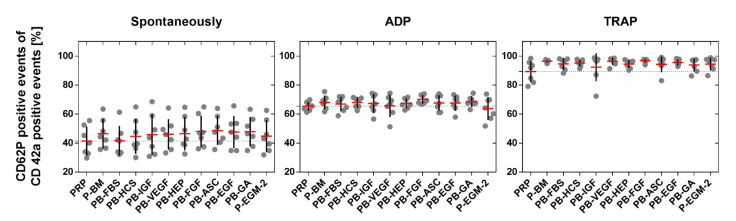
Amount of activated platelets. Platelet-rich plasma (PRP) was mixed with basal medium (BM) or with BM containing one of nine different supplements or with BM containing all nine supplements (see Table 1 for nomenclature) in a ratio of 1:1. PRP served as control. PLT were incubated for 5 min with different supplements (spontaneous activation) or with supplements and a platelet activator (adenosine diphosphate (ADP) or thrombin receptor activating peptide (TRAP)) prior to fixation and immunological staining to detect the surface markers CD42a (platelet marker) and CD62P (platelet activation marker) by flow cytometry. Thin dashed line shows the value for PRP (control). Data represent the arithmetic mean (red) ± standard deviation (black), *n* = 7.

The surface antigen type CD62P, which is solely expressed on activated platelets, and CD42a, which is present on platelets regardless of the activation state, were detected by flow cytometry in order to assess platelet activation in response to endothelial cell medium supplements. The addition of the platelet activators adenosine diphosphate (ADP) and thrombin receptor activating peptide (TRAP) as positive controls significantly increased the amount of activated PLT (ADP: 67 ± 3%; *p* ≤ 0.0012 and TRAP: 95 ± 2%; *p* ≤ 0.0003) compared to spontaneously activated PLT (46 ± 11%). However, no significant differences were detected between single medium supplements and PRP in none of the three tested groups (Figure 5) indicating that none of the tested medium components affect PLT activation. For more detailed information on flow cytometry data such as dot plots and gating, the reader is referred to Supplementary Figure S3. 

### 2.3. Platelet Aggregation

**Figure 6:**
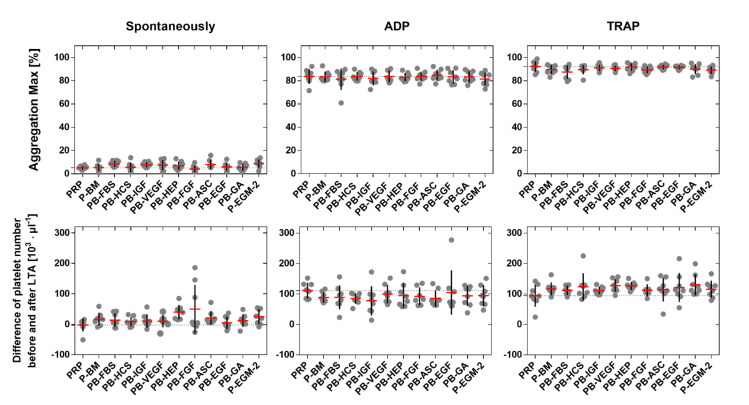
Aggregation of platelets. Platelet-rich plasma (PRP) was mixed with basal medium (BM) or with BM containing one of nine different supplements or with BM containing all nine supplements (see Table 1 for nomenclature) in a ratio of 1:1. PRP served as control. PLT were incubated for 10 min under rotation with different supplements (spontaneous aggregation) or in the presence of activators (adenosine diphosphate (ADP) or thrombin receptor activating peptide (TRAP)) prior to the assessment of the maximal aggregation by light transmission aggregometry (LTA) (first row). The difference of PLT number before and after LTA was calculated to indirectly assess PLT aggregation (second row). Thin dashed line shows the maximal aggregation for PRP (control). Data represent the arithmetic mean (red) ± standard deviation (black), *n* = 8.

PLT were incubated with each supplement and the maximal aggregation was recorded by light transmission aggregometry (LTA) in order to evaluate the effect of different EC medium compositions on platelet aggregation. Pure PRP served as control. Generally, the spontaneous maximal aggregation was significantly lower (7 ± 2%) than the maximal aggregation induced by ADP (83 ± 4%; *p* ≤ 0.0363) and TRAP (90 ± 2%; *p* ≤ 0.0002). However, no significant differences were found between single medium supplements and PRP in none of the three tested groups (Figure 6, first row). The same was found when the number of PLT was counted before and after LTA, which is an indirect method for the analysis of PLT aggregation. The difference in PLT number was significantly higher after activation with ADP (94 ± 31 PLT µL^−1^; *p* ≤ 0.0344) and TRAP (117 ± 22 PLT µL^−1^; *p* ≤ 0.0006) compared to the spontaneous activation (18 ± 12 PLT µL^−1^). However, no significant differences were found between single medium supplements and PRP within each of the three tested groups (Figure 6, second row), indicating that none of the tested medium components affect PLT aggregation. 

## 3. Discussion

The assessment of the thrombogenic potential of polymer-based biomaterials in the presence of both EC and PLT under near-physiological conditions is still a major challenge. In principle, two types of test systems for two different scenarios are possible: First, a test system that allows contact of a cardiovascular device with an endothelial cell monolayer on one side and with platelets-containing blood on the other side. Second, a test system that mimics the complete endothelialization of a cardiovascular device, which prevents the direct contact to blood and platelets. The latter test system requires co-cultivation of endothelial cells and platelets. One major challenge of such in vitro test systems became apparent in our previous study, which showed that EC detach in response to PLT [5]. Here, the influence of individual medium components was systematically analyzed with regard to PLT adherence to two different polymeric biomaterials such as plasma-treated COC and PTFE, PLT activation, and aggregation. PLT adherence was rather polymer- and donor-dependent than supplement-dependent. While on COC, most PLT exhibited a discoid morphology, they showed numerous stages of activation such as widely spread PLT and pseudopodia formation on PTFE, independent of the supplements. Quantitative data revealed that supplements lead to increased or decreased areas per PLT, PLT covered areas, and numbers of adherent PLT compared to the control depending on the polymer. It is firmly established that PTFE respectively structurally modified, expanded PTFE (ePTFE; the predominant form of PTFE used for vascular grafts) show increased thrombogenicity when compared to biological vessels. For example, in patients requiring vascular access for hemodialysis, the patients own vessels were superior to PTFE and all other tested materials [12,13]. With regard to above-the-knee femoro-popliteal bypasses, autologous vein grafts resulted in significantly higher patency rates than PTFE [14]. Also, among synthetic materials, the high thrombogenicity of PTFE is undisputed. A 6-year prospective study comparing the use of poly(ethylene terephthalate) (PET) and ePTFE as y-aortic bifurcation grafts found no significant differences between the two tested materials in terms of long-term patency. However, PTFE grafts were associated with a higher rate of complications [15]. Polyurethane (PU)-based vascular grafts confer a better compliance to the native vasculature; however, graft patency rates compared to PTFE and PET are equally poor [16]. The same result was found for above-the-knee femoropopliteal bypass grafts. The two-year primary patency rate was higher for PET than for PTFE suggesting that PET is even superior to PTFE [17]. In hemocompatibility studies in vitro, PTFE is often used as a positive control to demonstrate the functionality of PLT in terms of adherence and aggregation [18]. Despite the thrombogenic potential of PTFE, the lack of alternative materials make it the material of choice when autologous vessels are not available [19]. 

COC are mainly used for biological microfluidic devices and cell culture substrates suitable for microscopy due to their excellent optical properties; however, they are also increasingly discussed as implant materials [20]. Within the scope of establishing an in vitro thrombogenicity test setup, we have previously analyzed the physicochemical properties of the plasma-treated COC-based slide as part of a commercial perfusion system [21]. In this application, the COC-based substrate serves as a structural support for the formation of a functional-confluent EC monolayer. Here, we investigated pCOC regarding PLT adherence in response to individual medium supplements. The data revealed that pCOC has a weak thrombogenic potential regardless of the presence of any supplement. This was also shown in a study, which assessed the hemocompatibility of multiple types of COC. In particular, fewer PLT adhered to all types of COC compared to glass but P-selectin (CD62P) expression, a marker for PLT activation, was detected. Moreover, dynamic blood coagulation assays revealed an accelerated aggregation of PLT on COC compared to a control containing no material [20]. 

Apart from the polymer-dependent PLT adherence, a high variability in the number of adherent PLT and the PLT covered area was observed for PLT of individual donors on PTFE. Donor variability affecting cell differentiation and other diverse cellular responses is documented in various research areas such as tissue engineering or transfusion medicine [22,23]. This is particularly important for autologous therapies as patients (and their cells) are often of advanced age. Thus, studies increasingly use age-matched cells or animals to better reflect the situation in this group of patients [24]. The data of the present study confirm once more that inter-donor variability exists and that the hemocompatibility of biomaterials should be analyzed either individually for each recipient in a personalized approach or at least using PLT of a substantial number of donors. 

With regard to PLT activation and aggregation, no effect of either of the tested supplements was detected. This disproves the hypothesis that specific endothelial cell medium components might induce PLT activation and result in EC detachment as has been observed in co-cultures [5]. Another possible cause for this phenomenon might have been the static culture conditions, which do not reflect the physiological situation. Recently, we showed that laminar flow promotes EC growth and EC did not detach in response to PLT in the presence of complete EGM-2 medium, neither under dynamic nor static conditions [21]. We therefore hypothesize that other factors such as the substrate (glass) were responsible for this phenomenon. Glass has been the material of choice in the very beginning of cell culture [25]. However, it was soon observed that many cell types struggled with adhesion and proliferation on glass. Today, plasma-treated polymers such as COC or substrates coated with proteins are preferentially used [26].

## 4. Materials and Methods

### 4.1. Polymeric Substrates

Adherence of platelets to two different polymeric substrates was investigated using six-channel µ-Slides 0.4 (ibiTreat, Ibidi, Gräfelfing, Bavaria, Germany) and six-channel Sticky-Slides VI µ-Slides 0.4 (Ibidi), respectively. Six-channel µ-Slides 0.4 are composed of a plasma-treated cyclic olefin copolymer (pCOC)-based substrate used for cell adherence and a cyclic olefin polymer (COP)-based upper part. pCOC exhibits a low roughness and a moderate hydrophilicity (*R*_q_ = 1.1 ± 0.5 nm, *θ*_adv_ = 62 ± 1° [21]). Six-channel Sticky-Slides VI µ-Slides 0.4 are composed of an upper COP-based part and can be attached to any desired material. Here, it was attached to PTFE (Bess pro GmbH, Berlin, Germany), which was used as a positive control for platelet adherence. PTFE also exhibits a smooth but a hydrophobic surface (*R*_q_ = 62 ± 6 nm, *θ*_adv_ = 119 ± 2°) [27].

### 4.2. Characterization of Whole Blood 

Whole blood anticoagulated with trisodium citrate (German Red Cross, Berlin, Germany) of 14 donors (13 male/1 female; 27–64 years resp. 47 ± 12 years) were used for experiments in accordance with the ethics committee of Charité University Medicine Berlin (EA2/012/10). Citrated whole blood was allowed to rest for 15 min prior to experiments. To exclude blood anomalies regarding platelet function, the blood was subjected to a quality test according to the guidelines of the Nordkem workshop [28]. For this, blood was analyzed using the hematology analyzer Sysmex XS-800i (Fluorescence Flow Cytometer, Norderstedt, Germany) regarding standard hemogram data including number of red- and white blood cells as well as platelets, mean erythrocyte- and platelet volume, hemoglobin and hematocrit. Moreover, platelet function was tested as a function of closure time in a PFA-100^®^ system (Platelet Function Analyzer, Siemens Healthcare Diagnostics, Marburg, Germany). C-reactive protein levels were semi-quantitatively assessed to exclude inflammation (IMACO, Lüdersdorf, Germany). Subjects were excluded when values were not within the reference ranges for healthy humans. 

### 4.3. Isolation of Platelet-Rich Plasma (PRP) and Platelet-Poor Plasma (PPP) from Whole Blood

Platelet-rich plasma (PRP) was isolated by centrifugation of whole blood at 140× *g* for 20 min at 22 °C (Centrifuge 5804 R, Heraeus, Hanau, Germany). Platelet-poor plasma (PPP) was obtained by centrifugation at 1500× *g* for 30 min at 22 °C and used to adjust the target concentration of PRP. The number of PLT was counted using a hematology analyzer Sysmex XS-800i.

### 4.4. Preparation of Endothelial Cell Culture Media

EGM-2 medium consisted of a basal medium (BM) and nine different supplements listed in Table 1. To analyze the influence of each supplement on platelet adherence, activation and aggregation, PRP was mixed in a ratio of 1:1 (by volume) with BM or with BM containing one of each of the nine supplements or with BM containing all nine supplements (complete medium, P-EGM-2). Each supplement was applied in the concentration specified by the manufacturer of EGM-2 (Lonza, Cologne, Germany). PRP served as a control. Thus, 12 different compositions were tested. 

### 4.5. Adherence of Platelets

To investigate the influence of different medium compositions on PLT adherence to two different polymeric substrates, PRP was isolated from six different donors (*n* = 6, all male, 27–56 years, 41 ± 11 years). PRP was either adjusted to 50,000 PLT µL^−1^ with PPP (for PRP control) or to 100,000 PLT µL^−1^ and further diluted in a ratio of 1:1 with each of the different medium compositions listed in Table 1 resulting in a final concentration of 50,000 PLT µL^−1^. Adherence tests were performed using six-channel µ-Slides VI 0.4 equipped with pCOC as substrate material or six-channel Sticky-Slides VI 0.4 with PTFE as a substrate as described above. Each of the six channels per slide was filled with 60 µL sample and incubated for 1 h at 37 °C. After incubation, channels were rinsed with phosphate buffered saline (PBS, Gibco, Paisley, UK) to remove non-adherent PLT and adherent PLT were fixed with 4% (*w*/*v*) paraformaldehyde (PFA, Sigma-Aldrich, Steinheim, Germany) for 30 min at 4 °C. After washing, PLT were simultaneously permeabilized and labelled with phalloidin conjugated to Alexa Fluor 555 (Invitrogen, OR, USA, A34055, 1:40) in PBS containing 2% (*w*/*v*) bovine serum albumin (BSA, Roth, Karlsruhe, Germany) and 0.3% (*v*/*v*) Triton-X-100 (Roth). After 1 h of incubation at room temperature (RT) in the dark, PLT were washed with PBS and mounted with mowiol (Roth) and cover slips (# 1.5, Menzel GmbH, Braunschweig, Germany). While pCOC-slides were directly covered with mowiol, PTFE was removed from the Sticky-Slides and transferred to conventional glass slides prior to mounting with mowiol and cover slips. Staining was performed in duplicates (two channels per sample) for each donor. Dried samples were analyzed by confocal laser scan microscopy (DMi8 TCS SP8 STED, Leica Microsystems CMS GmbH, Wetzlar, Germany). In total, 1728 images were taken (six independent experiments with 11 different medium compositions and PRP as a control performed in duplicates on two different polymers whereas six images were taken per channel). 

### 4.6. Activation of Platelets

To investigate the influence of different medium compositions on PLT activation, PRP of seven different donors (*n* = 7, 6 male/1 female, 35–64 years, 51 ± 10 years) was mixed with each of the medium compositions listed in Table 1 in a ratio of 1:1 both in the absence or presence of adenosine diphosphate (ADP, final concentration 0.02 mM) or thrombin receptor activating peptide (TRAP, final concentration 0.1 mM) as positive controls. Samples were incubated for 5 min at RT prior to fixation with 100 µL ThromboFix^TM^ Platelet Stabilizer (Beckman Coulter, Fullerton, USA) for 1 h at RT. To reduce unspecific binding of antibodies, 500 µL PBS containing 1% (*w*/*v*) BSA was added and incubated overnight at 4 °C. The surface-bound activation markers CD42a and CD62P were immunologically stained with the following antibodies: mouse anti-human CD42a coupled to FITC (Becton Dickinson, San Jose, CA, USA, 348083, 1:32) and mouse anti-human CD62P coupled to PE (Becton Dickinson, San Jose, CA, USA, 348107, 1:32) in PBS containing 1% (*w*/*v*) BSA. In addition, single stained PLT and unstained PLT were prepared for spillover compensation. Samples were incubated for 30 min at 4 °C in the dark prior to the addition of 500 µL MACS Running Buffer (Miltenyi Biotech, Bergisch Gladbach, Germany) and flow cytometric analysis. Generally, 15,000 events per sample were recorded while events are defined as every recognized signal representing both single PLT and PLT aggregates. Data analysis was performed using the software MACSQuantify^TM^ (Version 2.11). 

### 4.7. Aggregation Assays of Platelets

#### 4.7.1. Light Transmission Aggregometry 

To assess the spontaneous platelet aggregation after contact with different EC medium compositions, 90 µL PRP from eight different donors (the same as used for activation studies plus one additional donor, *n* = 8, 7 male/1 female, 35–69 years, 53 ± 11 years) was mixed with 90 µL of each of the 11 compositions listed in Table 1 and filled in a cuvette. While the spontaneous aggregation was measured after the addition of 20 µL PPP, agonist-induced aggregation was measured after the addition of 20 µL ADP (final concentration 0.02 mM) or TRAP (final concentration 0.1 mM). PLT aggregation was assessed by light transmission over a period of 10 min at 37 °C and was expressed as maximal aggregation in percent. While non-aggregated PLT show maximal turbidity, PLT aggregates clear up the cell suspension. Thus, calibration of the device was performed with 180 µL PPP for 0% turbidity and 180 µL PRP for 100% turbidity. 

#### 4.7.2. Platelet Count

An indirect method to analyze platelet aggregation is to measure the difference in PLT number before and after light transmission aggregometry. This was done by the Sysmex XS-800i Analyzer, which is based on a flow cytometric principle. While single PLT are easily measured by the device, large aggregates are not recognized as platelets and thus are not counted leading to low numbers of PLT. Thus, a high difference of PLT count before and after LTA measurement corresponds to increased aggregation. 

#### 4.7.3. Statistics

Statistical analyses were performed using Graphpad Prism 6 (Graphpad Software, San Diego, CA, USA). Gaussian distribution of the data was tested using the Kolmogorov-Smirnov test. Data are reported as arithmetic mean ± standard deviation. Comparisons of non-parametric data between multiple groups were performed by Kruskal–Wallis test and Dunn’s posttest, and between two groups by Mann–Whitney test. Differences were considered significant at *p* ≤ 0.05. 

## 5. Conclusions

Other than initially hypothesized, individual endothelial medium supplements do not lead to PLT activation and aggregation. In contrast, PLT adhesion was differently affected, although in a polymer- and donor-dependent manner, which is an important finding for the establishment of near-physiological in vitro thrombogenicity test systems. Future studies will establish a test system using complete EGM-2 medium containing all nine supplements to evaluate different endothelialized polymers in contact with PLT regarding their thrombogenic potential.

## Figures and Tables

**Table 1:** Compositions of endothelial cell culture media mixed with platelet-rich plasma (PRP).

	Medium Composition	Sample ID
**1**	PRP + Basal medium (BM)	P-BM
**2**	PRP + BM + fetal bovine serum (FBS)	PB-FBS
**3**	PRP + BM + hydrocortisone (HCS)	PB-HCS
**4**	PRP + BM + insulin-like growth factor-1 (IGF-1)	PB-IGF-1
**5**	PRP + BM + vascular endothelial growth factor (VEGF)	PB-VEGF
**6**	PRP + BM + heparin (HEP)	PB-HEP
**7**	PRP + BM + human fibroblast growth factor (FGF)	PB-FGF
**8**	PRP + BM + ascorbic acid (ASC)	PB-ASC
**9**	PRP + BM + human epidermal growth factor (EGF)	PB-EGF
**10**	PRP + BM + gentamycin / amphotericin B (GA)	PB-GA
**11**	PRP + BM + FBS + HCS + IGF-1 + VEGF + HEP + FGF + ASC + EGF + GA	P-EGM-2

## Data Availability

The datasets generated during and/or analyzed during the current study are available from the corresponding author on reasonable request.

## Supplementary Materials

The following are available online at https://www.mdpi.com/article/10.3390/ijms22137006/s1, Figure S1: Adherence of platelets (PLT) on plasma-treated cyclic olefin copolymer, Figure S2: Adherence of platelets (PLT) on poly(tetrafluoroethylene), Figure S3: Quantification of platelet activation.
