# Supplementary material for: Effect of Endothelial Culture Medium Composition on Platelet Responses to Polymeric Biomaterials
Source: Int J Mol Sci. 2021 Jun 29;22(13):7006. doi: 10.3390/ijms22137006 (PMC8268423; doi:10.3390/ijms22137006)
Supplement: Supplementary file 1 [file ijms-22-07006-s001.zip › ijms-1262937-supplementary.pdf]

Supplementary information for

Effect of Endothelial Culture Medium Composition on Platelet Responses to Polymeric Biomaterials

Skadi Lau[1#], Anna Maier[1#], Steffen Braune[1], Manfred Gossen[1], Andreas Lendlein[1,2,*]

1 Institute of Active Polymers and Berlin-Brandenburg Center for Regenerative Therapies, Helmholtz-Zentrum Hereon, 14513 Teltow, Germany
2 Institute of Chemistry, University of Potsdam, 14476 Potsdam, Germany
# These authors contributed equally to this work
* Correspondence: andreas.lendlein@hereon.de

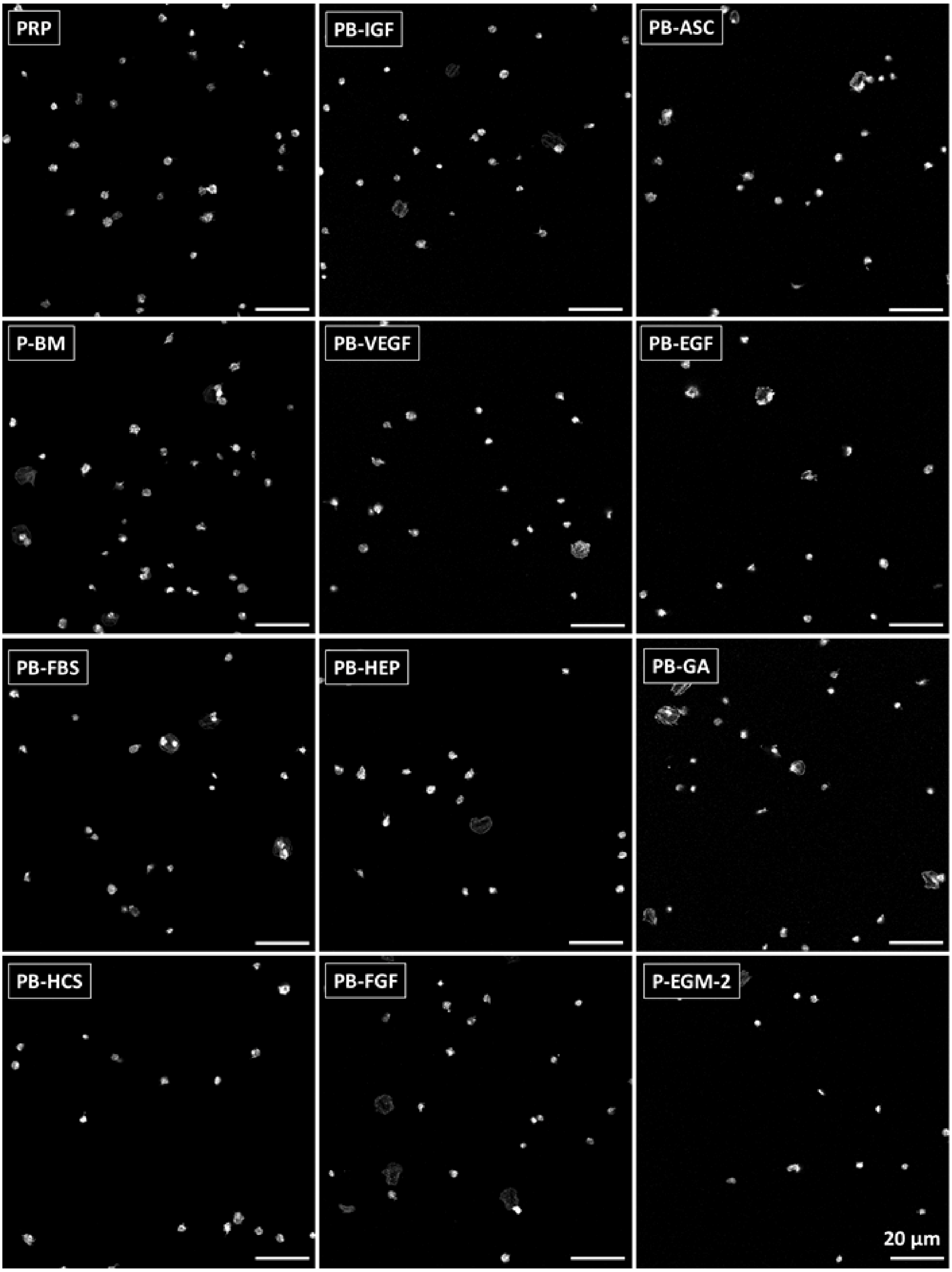

Supplementary figure 1: Adherence of platelets (PLT) on plasma-treated cyclic olefin copolymer. Platelet-rich plasma (PRP) was incubated under static conditions for 1 h at 37 °C with basal medium (BM) or with BM containing one of nine different supplements or with BM containing all nine supplements (see Table 1 for nomenclature) in a ratio of 1:1 resulting in a concentration of 50,000 PLT $\cdot$ μL^{-1}. PRP served as control. PLT of six different donors were stained with phalloidin conjugated with Alexa Fluor 555 and images were taken by confocal laser scan microscopy at 100-fold primary magnification. As platelet adherence was similar for all six donors and for all supplements tested, representative images of adherent PLT after contact with each of the tested supplements are shown. Scale bar represents 20 µm.

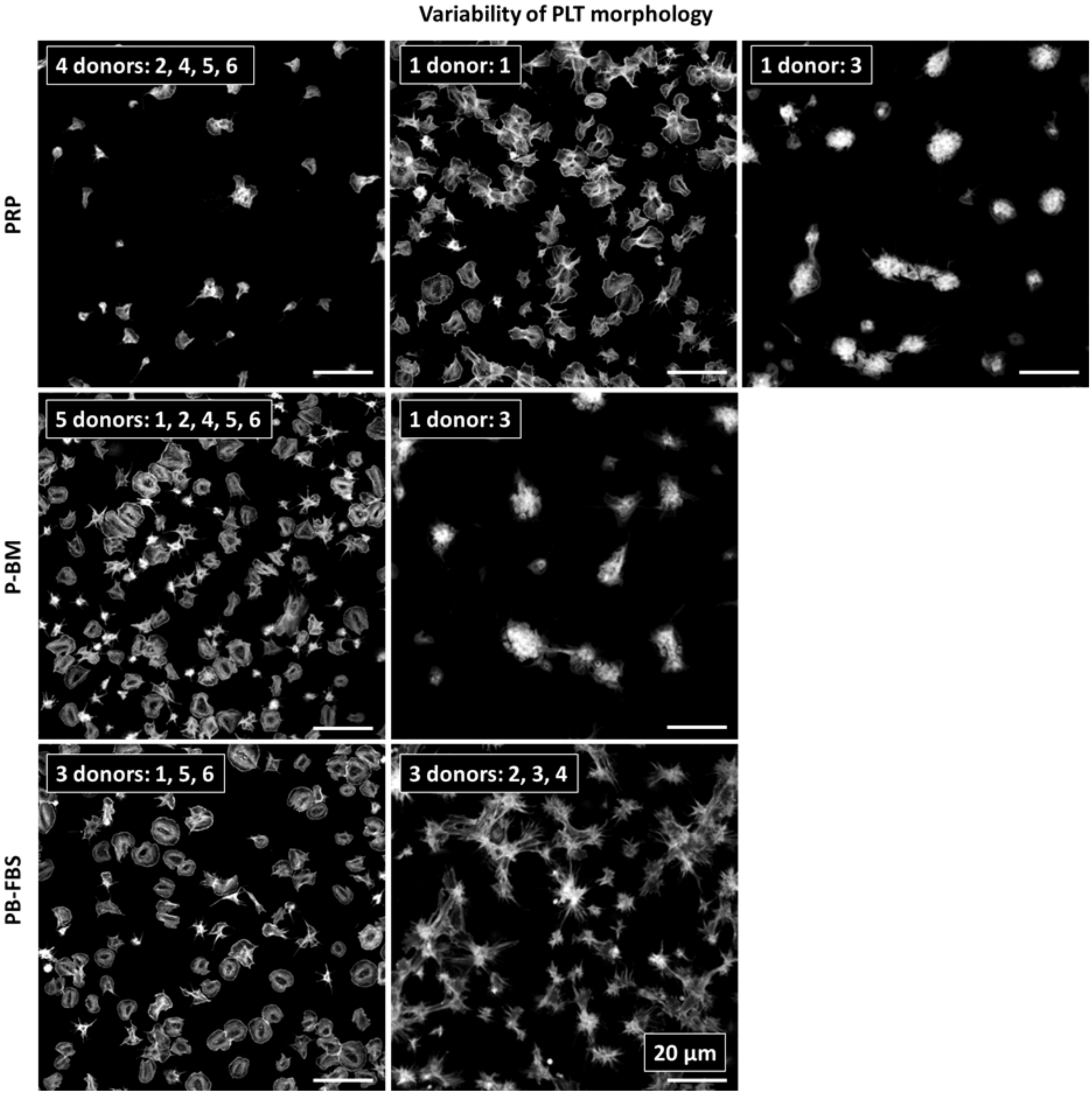

Supplementary figure 2: Adherence of platelets (PLT) on poly(tetrafluoro ethylene). Platelet-rich plasma (PRP) was incubated under static conditions for 1 h at 37 °C without or with basal medium (P-BM) or with BM containing fetal bovine serum (PB-FBS) in a ratio of 1:1 resulting in a concentration of 50,000 PLT · μL^{-1}. PRP served as control. PLT of six different donors (numbered from 1-6) were stained with phalloidin conjugated with Alexa Fluor 555 and images were taken by confocal laser scan microscopy at 100-fold primary magnification. Shown are different classes of platelet morphologies (with the individual donors representing them as indicated) after incubation with each supplement tested. Up to five different PLT morphologies depending on the donor are shown to illustrate the high donor variability. Scale bar represents 20 µm.

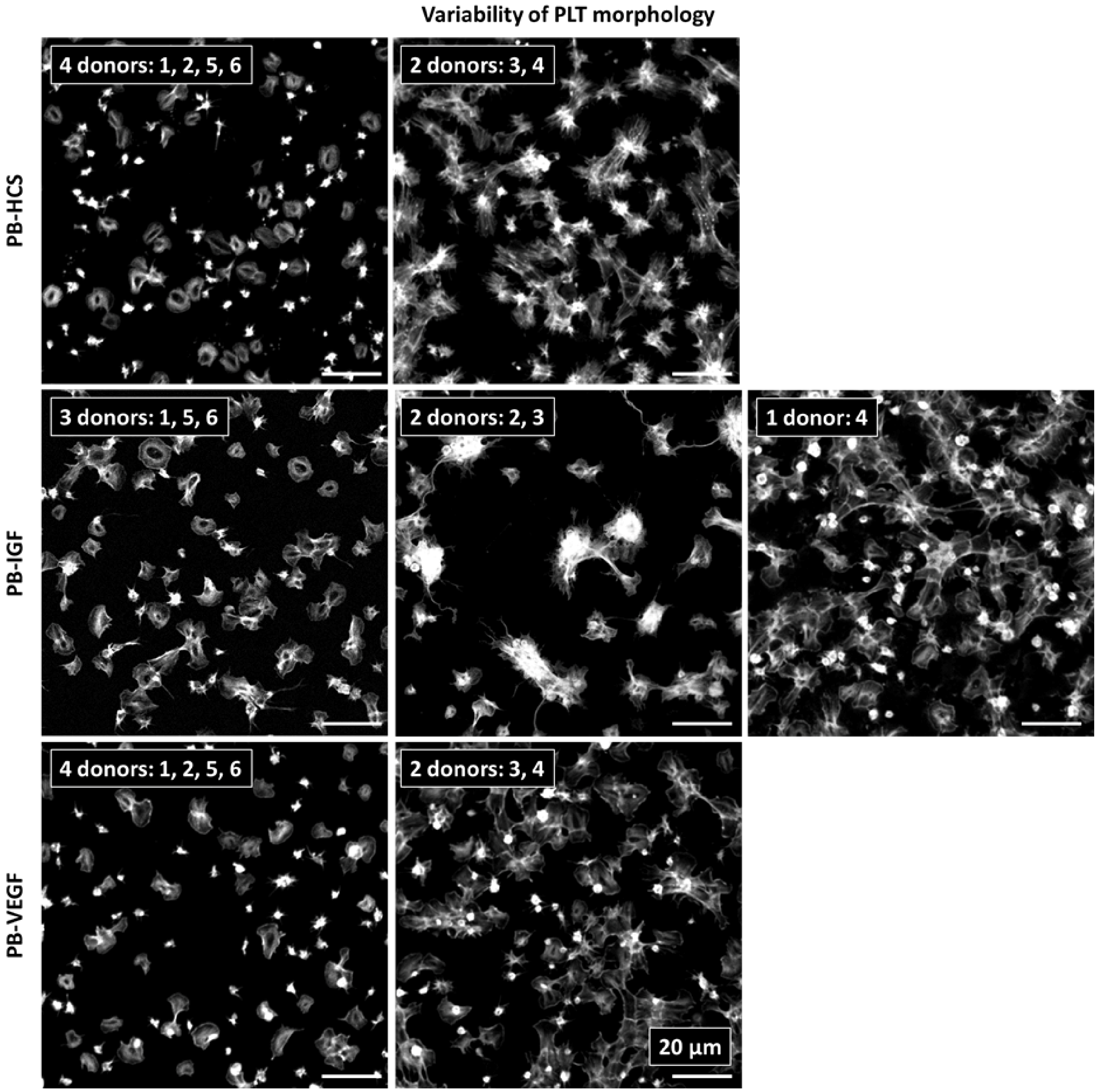

Supplementary figure 2 (continued): Adherence of platelets (PLT) on poly(tetrafluoro ethylene). Platelet-rich plasma (PRP) was incubated under static conditions for 1 h at 37 °C with basal medium (BM) containing hydrocortisone (PB-HCS), insulin-like growth factor-1 (PB-IGF) or vascular endothelial growth factor (PB-VEGF) in a ratio of 1:1 resulting in a concentration of 50,000 PLT · μL^{-1}. PRP served as control. PLT of six different donors (numbered from 1-6) were stained with phalloidin conjugated with Alexa Fluor 555 and images were taken by confocal laser scan microscopy at 100-fold primary magnification. Shown are different classes of platelet morphologies (with the individual donors representing them as indicated) after incubation with each supplement tested. Up to five different PLT morphologies depending on the donor are shown to illustrate the high donor variability. Scale bar represents 20 µm.

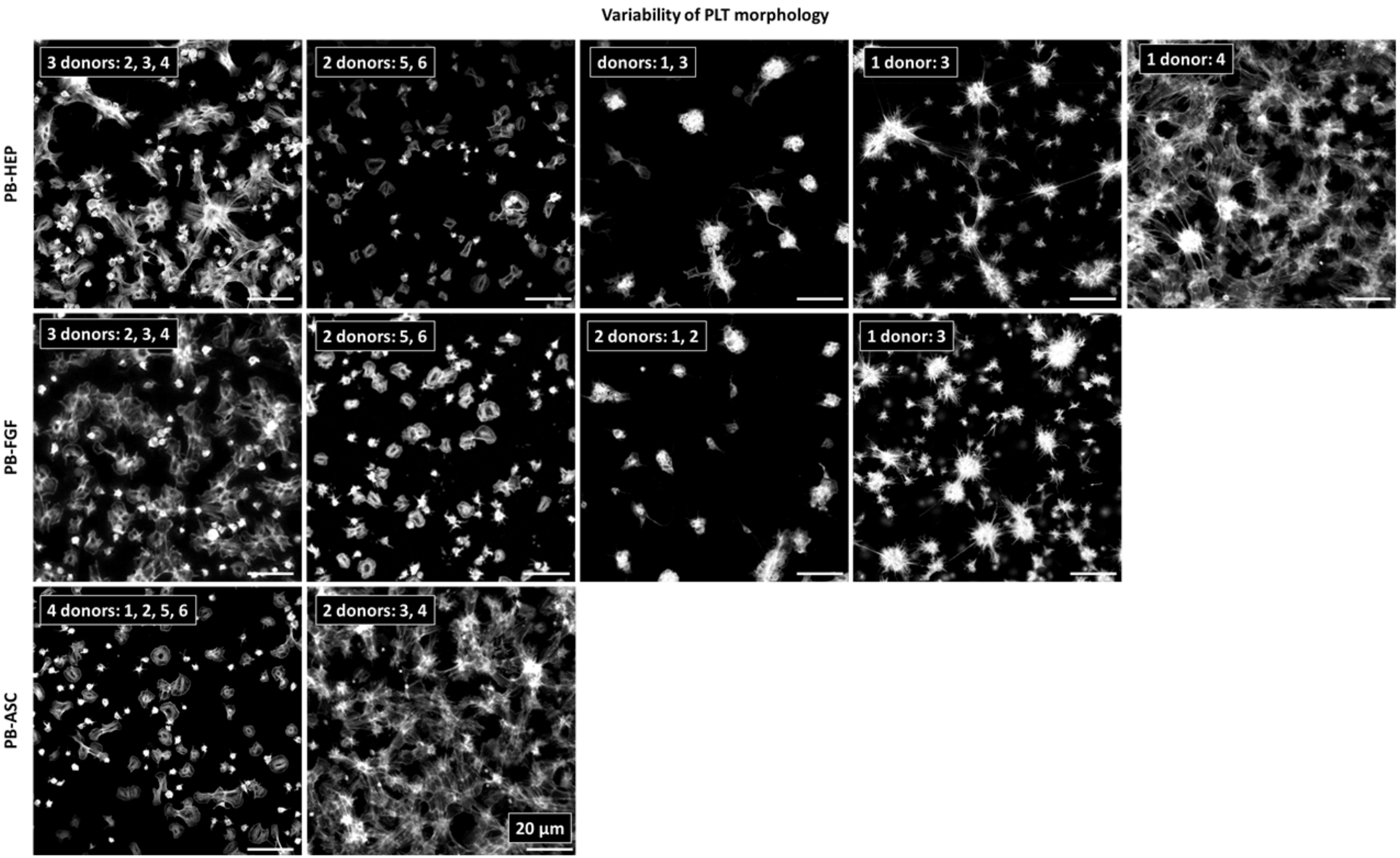

Supplementary figure 2 (continued): Adherence of platelets (PLT) on poly(tetrafluoro ethylene). Platelet-rich plasma (PRP) was incubated under static conditions for 1 h at 37 °C with basal medium (BM) containing heparin (PB-HEP), human fibroblast growth factor (PB-FGF) or ascorbic acid (PB-ASC) in a ratio of 1:1 resulting in a concentration of 50,000 PLT · μL^{-1}. PRP served as control. PLT of six different donors (numbered from 1-6) were stained with phalloidin conjugated with Alexa Fluor 555 and images were taken by confocal laser scan microscopy at 100-fold primary magnification. Shown are different classes of platelet morphologies (with the individual donors representing them as indicated) after incubation with each supplement tested. Up to five different PLT morphologies depending on the donor are shown to illustrate the high donor variability. Scale bar represents 20 µm.

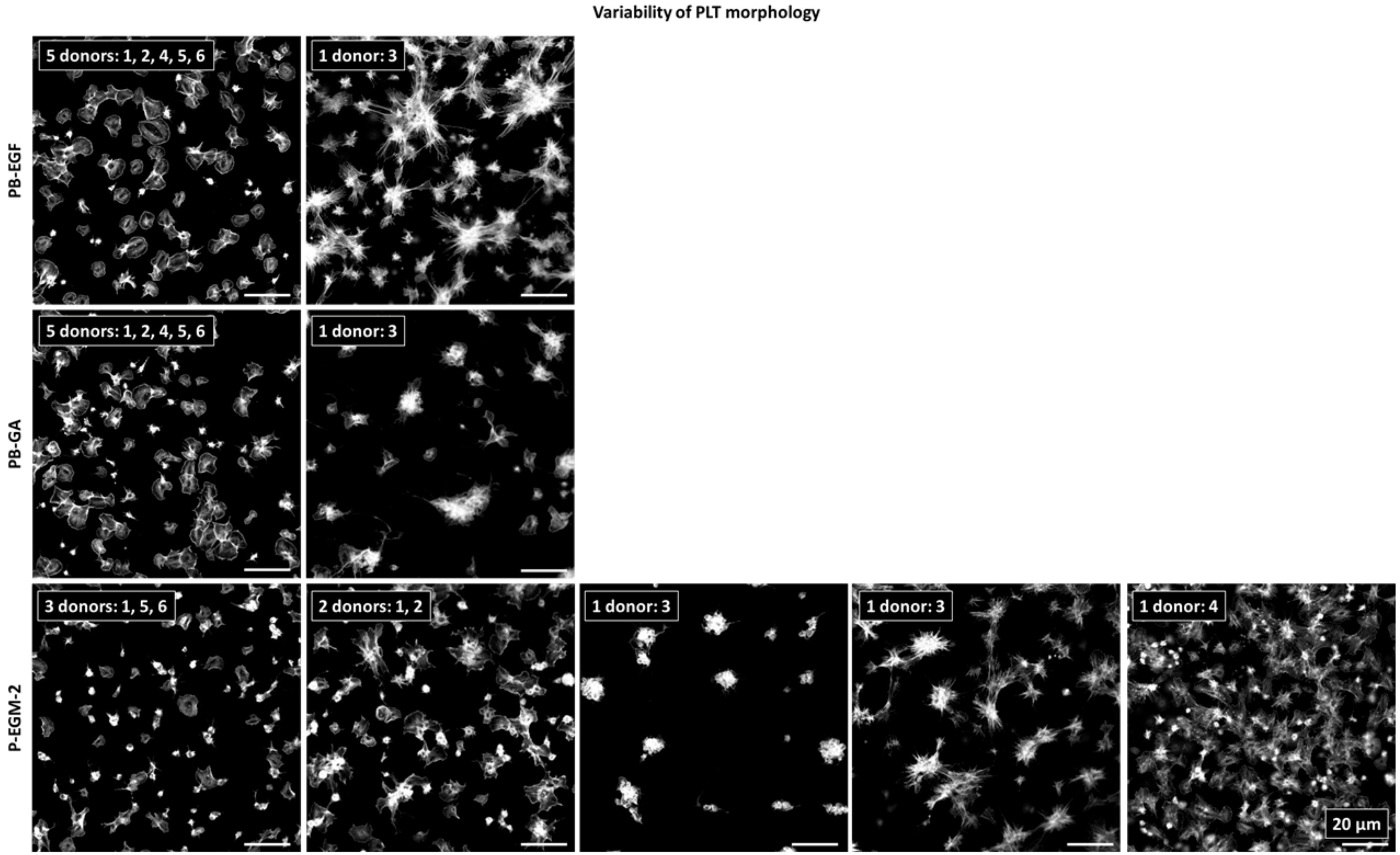

Supplementary figure 2 (continued): Adherence of platelets (PLT) on poly(tetrafluoro ethylene). Platelet-rich plasma (PRP) was incubated under static conditions for 1 h at 37 °C with basal medium (BM) containing human epidermal growth factor (PB-EGF), gentamycin and amphotericin B (PB-GA) or complete EGM-2 medium (P-EGM-2) in a ratio of 1:1 resulting in a concentration of 50,000 PLT · μL^{-1}. PRP served as control. PLT of six different donors (numbered from 1-6) were stained with phalloidin conjugated with Alexa Fluor 555 and images were taken by confocal laser scan microscopy at 100-fold primary magnification. Shown are different classes of platelet morphologies (with the individual donors representing them as indicated) after incubation with each supplement tested. Up to five different PLT morphologies depending on the donor are shown to illustrate the high donor variability. Scale bar represents 20 µm.

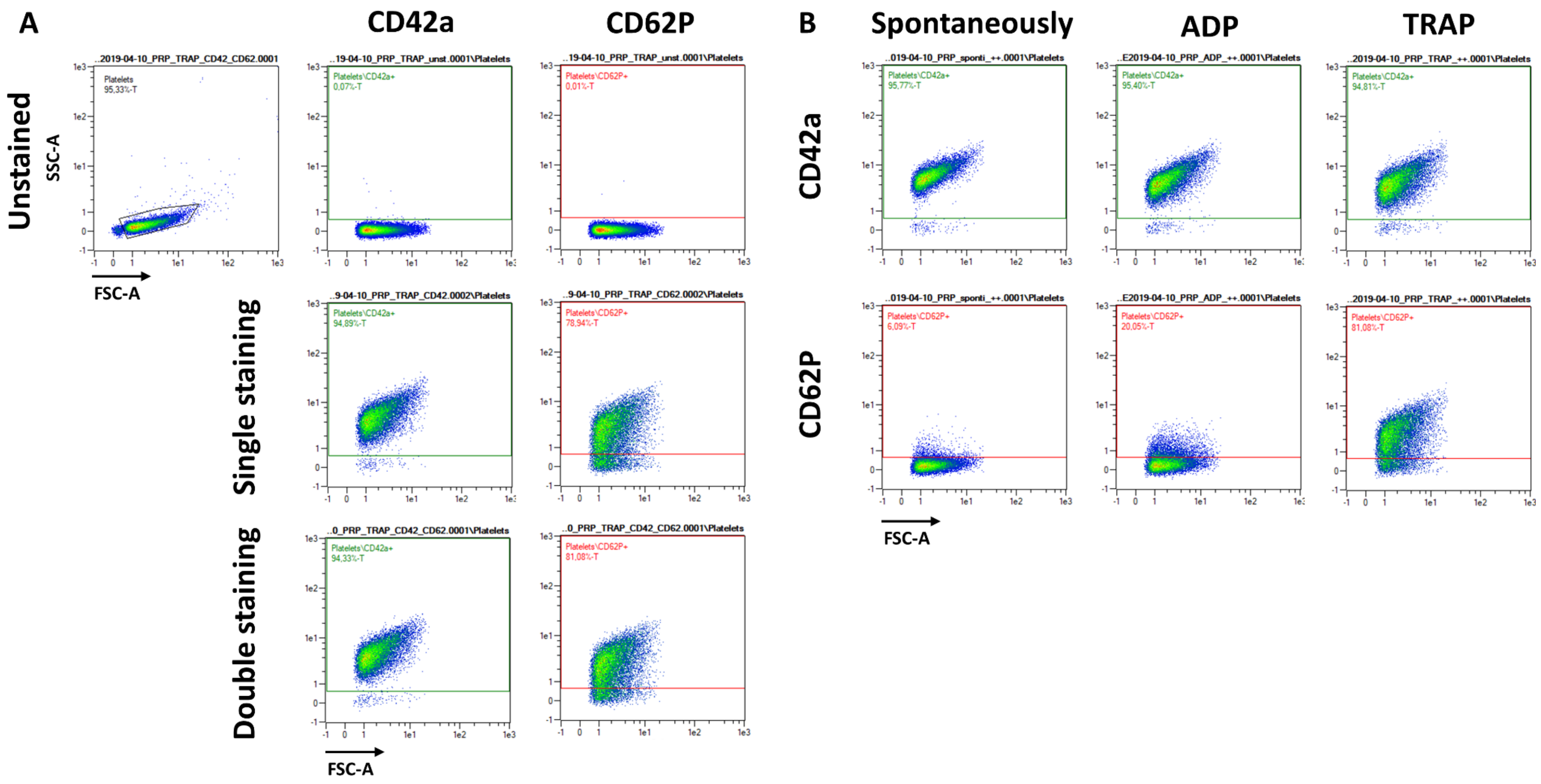

Supplementary figure 3: Quantification of platelet activation. Platelet-rich plasma (PRP) was mixed with basal medium (BM) or with BM containing one of nine different supplements or with BM containing all nine supplements (see Table 1 for nomenclature) in a ratio of 1:1. PRP served as control. PLT were incubated for 5 min with different supplements (spontaneous activation) or with supplements and an platelet activator (adenosine diphosphate (ADP) or thrombin receptor activating peptide (TRAP)) prior to fixation and immunological staining to detect the surface markers CD42a (platelet marker) and CD62P (platelet activation marker) by flow cytometry. Unstained cells were used to select the population of interest and to set gates for the discrimination of surface-marker-expressing and non-expressing cells. Single stained cells were used to compensate for fluorescent spillover. Double stained cells were used to quantify the expression of CD42a and CD62 (A). The percentage of PLT expressing CD42a and CD62 was quantified in response to individual supplements with or without ADP and TRAP (B). Data shown here refer to PRP; analogous analyses were performed for the 11 different medium compositions.
